# Pressurized intraperitoneal aerosol chemotherapy (PIPAC) with cisplatin and doxorubicin in combination with FOLFOX chemotherapy as a first-line treatment for gastric cancer patients with peritoneal metastases: single-arm phase II study

**DOI:** 10.1186/s12885-023-11549-z

**Published:** 2023-10-25

**Authors:** Martynas Luksta, Augustinas Bausys, Klaudija Bickaite, Rokas Rackauskas, Marius Paskonis, Raminta Luksaite-Lukste, Anastasija Ranceva, Rokas Stulpinas, Birute Brasiuniene, Edita Baltruskeviciene, Nadezda Lachej, Rasa Sabaliauskaite, Rimantas Bausys, Skaiste Tulyte, Kestutis Strupas

**Affiliations:** 1 Clinic of Gastroenterology, Nephrourology, and Surgery, Institute of Clinical Medicine, Faculty of Medicine, Vilnius University, Vilnius, Lithuania Ciurlionio str. 21, 03101; 2 Department of Abdominal Surgery and Oncology, National Cancer Institute, Vilnius, Lithuania; 3 Centre for Visceral Medicine and Translational Research, Faculty of Medicine, Institute of Clinical Medicine, Vilnius University, Vilnius, 03101 Lithuania; 4 Department of Radiology, Nuclear Medicine and Medical Physics, Institute of Biomedical Sciences, Faculty of Medicine, Vilnius University, Vilnius, Lithuania; 5 Hematology, Oncology, and Transfusion Medicine Center, Vilnius University Hospital Santaros Klinikos, Vilnius, Lithuania; 6 National Center of Pathology, Affiliate of Vilnius University Hospital Santaros Klinikos, Vilnius, Lithuania; 7 Department of Medical Oncology, National Cancer Institute, Vilnius, Lithuania; 8 Institute of Clinical Medicine, Faculty of Medicine, Vilnius University, Vilnius, Lithuania; 9 Laboratory of Genetic Diagnostic, National Cancer Institute, Santariškių 1, Vilnius, LT-08406 Lithuania

**Keywords:** Gastric cancer, Peritoneal metastases, PIPAC

## Abstract

**Background:**

Gastric cancer (GC) remains among the most common and most lethal cancers worldwide. Peritoneum is the most common site for distant dissemination. Standard treatment for GC peritoneal metastases (PM) is a systemic therapy, but treatment outcomes remain very poor, with median overall survival ranging between 3-9 months. Thus, novel treatment methods are necessary. Pressurized intraperitoneal aerosol chemotherapy (PIPAC) is the most novel technique for intraperitoneal chemotherapy. Some preliminary data suggest PIPAC can achieve improved long-term outcomes in patients with GC PM, especially when used in combination with systemic chemotherapy. However, there is a lack of data from well-design prospective studies that would confirm the efficacy of PIPAC and systemic therapy combination for first-line treatment.

**Methods:**

This study is an investigator-initiated single-arm, phase II trial to investigate the efficacy of PIPAC combined with systemic FOLFOX (5-fluorouracil, oxaliplatin, leucovorin) as a first-line treatment for GC PM. The study is conducted in 2 specialized GC treatment centers in Lithuania. It enrolls GC patients with histologically confirmed PM without prior treatment. The treatment protocol consists of PIPAC with cisplatin (10.5 mg/m2 body surface in 150 mL NaCl 0.9%) and doxorubicin (2.1 mg/m2 in 50 mL NaCl 0.9%) followed by 2 cycles of FOLFOX every 6–7 weeks. In total 3 PIPACs and 6 cycles of FOLFOX will be utilized. The primary outcome of the study is the objective response rate (ORR) according to RECIST v. 1.1 criteria (Eisenhauer et al., Eur J Cancer 45:228–47) in a CT scan performed 7 days after the 4^th^ cycle of FOLFOX. Secondary outcomes include ORR after all experimental treatment, PIPAC characteristics, postoperative morbidity, histological and biochemical response, ascites volume, quality of life, overall survival, and toxicity.

**Discussion:**

This study aims to assess PIPAC and FOLFOX combination efficacy for previously untreated GC patients with PM.

**Trial registration:**

NCT05644249. Registered on December 9, 2022.

## Background

### Background and rationale

Gastric cancer (GC) is the 5th most common and 3rd most deadly cancer worldwide [1]. Peritoneal metastasis (PM), arising from GC, is the most common pattern of synchronous and metachronous dissemination and is generally associated with very poor long-term outcomes. Nowadays, the median survival of patients with GC PM ranges only between 2 and 9 months [2–6]. The standard treatment for GC PM is systemic chemotherapy alone or in combination with targeted therapy or immunotherapy. Although such treatment has very limited efficacy with only 14–25% of cases responding to it [7–9]. Several reasons are responsible for such limited efficacy. First, the plasma-peritoneal barrier isolates the peritoneum from the cytotoxic effect of intravenous chemotherapy. Second, poor intraperitoneal blood supply results in poor oxygenation of peritoneal cells, and this hypoxic state is associated with low apoptotic potential [10]. To overcome existing barriers intraperitoneal application of chemotherapy has been proposed. It offers pharmacokinetic advantages over intravenous therapy because high intraperitoneal drug concentration can be achieved while maintaining low systemic drug concentration, thus reducing treatment toxicity. Pressurized intraperitoneal aerosol chemotherapy (PIPAC) is the most novel technique for intraperitoneal chemotherapy. Through the procedure, special laparoscopic instruments are used to deliver drugs into the abdominal cavity as an aerosol under pressure. The rationale for PIPAC relies on physical and biological law which show that: (1) more homogenous drug distribution can be achieved by applying an aerosol compared to a liquid solution, (2) increased intraperitoneal hydrostatic pressure counteracts elevated interstitial fluid pressure within PM, (3) limited blood outflow at the drug application moment helps to increase intratumoral cytotoxic drug concentration and (4) the nature of the procedure allows to monitor and adjust the environmental parameters such as pH, temperature, electrostatic charge, and others for the best efficacy. Moreover, PIPAC can be applied repeatedly and biopsies can be taken during the procedure for objective assessment of tumor regression [11, 12]. PIPAC can be used as a single method for treatment (“unidirectional”) or in a “bidirectional” manner when it is combined with systemic chemotherapy [13]. The bidirectional approach seems rational because intravenously applied chemotherapy may improve subperitoneal drug accumulation and also treat circulating tumor cells and systemic micrometastases [14]. Such a bidirectional approach for GC patients with PM has been reported to be safe and feasible. Also, it seems effective as pathologic response is achieved in about 60% of patients and 1-year overall survival (OS) rate exceeds 50% [15–17]. However, these studies are small and inconclusive. There is a need for a prospective study to investigate this promising treatment - bidirectional PIPAC as a first-line treatment for GC patients with PM.

### Objective

This study aims to investigate PIPAC and systemic FOLFOX (5-fluorouracil, oxaliplatin, and leucovorin) chemotherapy efficacy as a first-line treatment for GC patients with PM.

### Trial design

This investigator-initiated study is designed as a single-arm phase II trial to investigate the efficacy of PIPAC in combination with FOLFOX to treat GC PM.

## Methods

### Study setting

The study will be conducted at two major gastrointestinal cancer treatment centers in Lithuania: National Cancer Institute and Vilnius University hospital Santaros Klinikos.

### Eligibility criteria criteria

The study will include GC patients with histologically confirmed PM scheduled for the first-line treatment if they meet all of the following inclusion criteria:

1. Histologically verified gastric adenocarcinoma (HER2 negative) with peritoneal metastases;
2. Age ≥ 18;
3. ECOG ≤ 1;
4. Patient willing to participate;
5. Patient is the candidate for 1st line FOLFOX palliative systemic chemotherapy.

Patients will be excluded if they meet the following criteria:

1. Extra-abdominal metastases;
2. Siewert I type gastroesophageal junction cancer;
3. Mechanical bowel obstruction;
4. Allergy to study drugs;
5. History of previous intraperitoneal chemotherapy;
6. Pregnancy of refusal for birth control at least 6 months post-study treatment.

### Taking informed consent procedure

Before performing any study-related procedures, written informed consent (IC) will be obtained from the patient by the study physician. Before the screening visit, all patients will have been worked up according to standard institutional protocols for patients with GC. These include esophagogastroduodenoscopy with biopsy; chest and abdominal computed tomography (CT); diagnostic laparoscopy with peritoneal lavage and biopsy for patients with ≥ cT2 GC without extra-abdominal metastases on CT scan. At the screening visit physician will provide the patient with information and details about a study and will answer all the questions that the patient has. After the patient indicates that he/she had enough time to consider participation and clearly expresses willingness to be included in the study physician and patient will sign the IC. A copy of the signed IC will be given to the patient.

### Additional consent provisions for collection and use of participant data and biological specimens

An option for permission to reuse clinical data and biological specimens collected through the study is included in the IC form.

### Intervention description

#### PIPAC procedure description

PIPAC will be performed under general anesthesia. To prevent surgical site infections all patients will receive antibiotic prophylaxis - a single dose of cefazoline (1.0 g) will be administered intravenously during the induction of anesthesia. The surgical procedure will start by entering the abdominal cavity using an open technique described by Hasson [18] and placing a 10 mm balloon trocar. After insufflating CO_2_ 12mmHg capnoperitoneum will be achieved and an additional 5 mm balloon trocar will be placed under video control. Then diagnostic laparoscopy will be performed: peritoneal carcinomatosis index (PCI) will be documented, multiple biopsies from metastatic foci will be taken and ascites will be removed to measure volume and for cytological examination. In case there are no ascites peritoneal lavage will be performed. Then CAPNOPEN© (Reger Medizintechnik, GmbH, Villingendorf, Germany) is connected to an intravenous high-pressure injector and inserted into the abdomen through the 10 mm access port. A 5 mm camera will be inserted through the other port keeping the tip of the CAPNOPEN© in view. A safety checklist will be performed to ensure there is no gas leakage. Injection parameters will be adjusted to a flow rate of 0.5 mL/s and a maximum upstream pressure of 200 psi in the high-pressure injector to generate the aerosol and drug application will start. After application of cisplatin (10.5 mg/m^2^ body surface in 150 mL NaCl 0.9%) and doxorubicin (2.1 mg/m^2^ in 50 mL NaCl 0.9%), the therapeutic capnoperitoneum of 12 mmHg will be maintained for next 30 min at a temperature of 37 °C. Then, the chemotherapy aerosol will be evacuated via a separate hospital air-waste system, trocars will be retracted and PIPAC finishes.

#### Systemic chemotherapy and further treatment

**Fig. 1:**
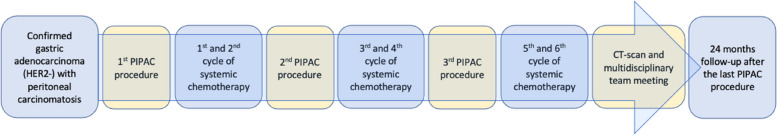
Patients treatment (standard systemic chemotherapy and PIPAC) pathway

Seven days after PIPAC patients will receive systemic chemotherapy. International guidelines recommend platinum-fluoropyrimidine doublet chemotherapy as a standard first-line chemotherapy for metastatic GC [19]. Thus, patients will receive FOLFOX chemotherapy which consists of intravenously administered folinic acid, 5-fluorouracil, and oxaliplatin. Within the next 4 weeks, 2 cycles of FOLFOX will be utilized. Then after 7–14 days of resting patients will again start treatment with PIPAC and the next 2 cycles of FOLFOX. In total 3 PIPACs and 6 cycles of FOLFOX will be utilized (Fig. 1).

##### Criteria for discontinuing or modifying allocated interventions

Patients can withdraw from the trial at any time by expressing their will to the study clinician. Also, different medical conditions may force them to discontinue or modify the study interventions. These include:

1. Mechanic bowel obstruction.
2. Intraabdominal adhesions that prevent safe access to the abdominal cavity.
3. Neutropenia defined by absolute neutrophil count < 1.5 × 10^^9^/L.
4. Thrombocytopenia: platelet count < 100 × 10^^9^/L.
5. Renal function insufficiency: by creatinine clearance < 50 ml/min.
6. Liver function insufficiency: AST/ALT > 3× the upper limit of normal or bilirubin > 2× the upper limit of normal.

Patients will be withdrawn from the study by the individual decision of the study clinician in consultation with the principal investigator.

###### Provisions for ancillary and post-trial care

After experimental treatment patients will undergo CT scans and further treatment will be discussed at multidisciplinary treatment meetings to offer an individual and best available treatment option for every patient.

### Outcomes

#### Primary outcome

The primary endpoint in this study is objective response rate (ORR) according to RECIST v. 1.1 criteria [20] in a CT scan performed 7 days after the 4th cycle of FOLFOX. ORR is the proportion of patients who have a complete response (CR), defined as the disappearance of all target lesions, or a partial response (PR), defined as ≥ 30% decrease in the sum of the diameters of target lesions.

#### Secondary outcomes

1. ORR according to RECIST v. 1.1 criteria in the CT-scan after all experimental treatment;
2. The median number PIPACs that can be utilized through the treatment protocol;
3. PIPAC characteristics (procedure time; intraoperative complications; length of a hospital stay after PIPAC; 30 day re-hospitalization rate);
4. Postoperative complications after PIPAC: assessed within 30 days after the PIPAC procedure and classified according to the Clavien-Dindo classification;
5. Peritoneal carcinomatosis index (PCI) measured at 2nd and 3rd PIPAC;
6. Histological regression of peritoneal metastases assessed by Peritoneal Regression Grading Score [21] measured in peritoneal biopsies at 2nd and 3rd PIPAC;
7. The volume of ascites measured at every PIPAC;
8. Biochemical tumor response: the concentration of carcinoembryonic antigen (CEA) and stomach cancer marker (Ca72-4);
9. Quality of life: it will be measured routinely using standard EORTC QLQ-C30 and EORTC QLQ-STO22 quality of life questionnaires;
10. Overall survival: defined as the time from the start of the treatment to study to death by any cause;
11. Progression-free survival: defined as the time from the start of the treatment to the progression of the disease diagnosed on CT scan or laparoscopy;
12. Toxicity according to the National Cancer Institute (NCI) Common Terminology Criteria (CTC) for adverse events v 5.0;
13. Biomarkers: gut microbiome composition, blood, and fecal biomarkers;

### Participant timeline

The participant timeline can be seen in Table 1.

**Table 1:**
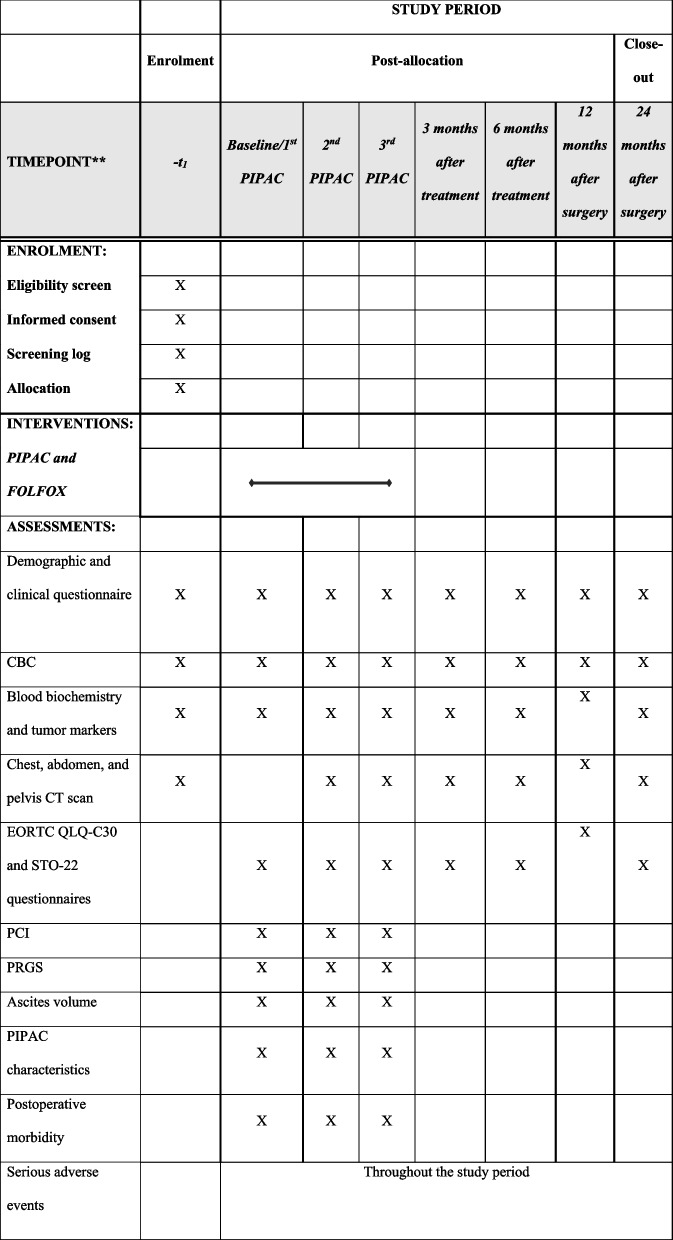
Participant timeline

### Sample size

In this study, we use Simon’s two-stage minimax design [22] (one-sided α 5% and power 80%). The response of conventional FOLFOX chemotherapy for GC PM is about 20% [23, 24]. Considering the side effects and tolerability of PIPAC combined with systemic FOLFOX chemotherapy, we thought that ORR increase to at least 40% is necessary as a clinically meaningful anti-tumor activity to proceed to a subsequent confirmatory trial. Thus, in the first stage of this study, 18 patients have to be enrolled. If ≤ 4 responses will be observed, the study will be terminated and declared negative. If at least five responses will be observed, an additional 15 patients will be accrued to the second stage. The study will meet its primary endpoint if confirmed responses will be observed in 11 or more patients out of a total of 33 response-evaluable patients. Considering the 10% dropout rate in total this study will include 37 patients.

### Recruitment

Participants will be recruited at 2 major gastrointestinal cancer treatment centers in Lithuania: National Cancer Institute and Vilnius University hospital Santaros Klinikos. The recruitment will be performed in the outpatient clinics by the clinicians who consult GC patients. All potentially eligible patients will be referred to a clinician-investigator who will screen if a patient meets the inclusion and does not meet exclusion criteria and will inform the patient about the clinical study. Patients willing to participate will be enrolled after signing written informed consent.

### Data collection

The data of participants will be collected according to the study protocol. Case report forms (CRFs) will be used to ensure the appropriate collection of necessary data. Routine auditing of the study documentation will be performed to ensure the quality of the data recorded in CRFs. To ensure the timeliness of the data, the CRFs will be completed within 3 working days following every visit. All data collected in CRFs will be transferred to an electronic database for further data collection and management. The confidentiality policy is outlined in an informed consent form and will be ensured during the data collection.

### Biological specimen collection

Peripheral venous blood samples and stool samples will be collected before the start of the treatment. Additionally, peritoneal metastases samples and ascites samples (100 ml) will be collected at the time of 1st PIPAC procedure.

All collected samples will be prepared according to standard laboratory protocols. Plasma and serum samples will be aliquoted in four 1ml tubes and stored at -80 C° in the laboratory at National Cancer Institute. Fresh stool samples will be split into four tubes containing at least 1 g of content and stored at -80 C° in the same laboratory. Gut microbiome analysis will be performed from stool samples by 16 S sequencing in the current trial. Also, biological specimens may be used for future studies.

### Statistical analysis

Accumulated data will be processed by SPSS (version 25) statistical software. All data will be checked for normality. Continuous variables will be expressed by mean with standard deviation or median with quartiles 1 and 3. Discrete variables will be expressed as proportions and percentages. Changes in the PCI, CEA, Ca72-4, and EORTC QLQ-C30 and EORTC QLQ-STO22 questionnaires score will be assessed by using paired sample t-test, Wilcoxon rank-sum test. For statistical analysis of gut microbiome compositions, the web-based application Calypso (version 8.84) will be used. Alpha diversity will be quantified by the Shannon index. Beta diversity will be quantified by principal coordinate analysis (PCoA) based on a Bray–Curtis dissimilarity matrix with analysis of similarity (ANOSIM), as well as redundancy analysis (RDA) with one or multiple explanatory variables. Additional analyses will be performed if necessary. P values < 0.05 will be considered statistically significant in all statistical analyses.

### Interim analyses

As mentioned previously this study is designed using Simon’s two-stage minimax design [22] (one-sided α 5% and power 80%). Thus, interim analysis will be performed after the first stage of the study when 18 patients will be enrolled. If ORR 7 days after the 2nd PIPAC will be achieved in ≤ 4 patients, the study will be terminated and declared negative. If at least five responses will be observed, an additional 15 patients will be accrued to the second stage.

#### Methods in analysis to handle protocol non-adherence and any statistical methods to handle missing data

Missing data will not be imputed.

### Plans to give access to the full protocol, participant-level data, and statistical code

Non-identifiable patient-level data will be available from the principal investigator upon reasonable request.

#### Composition of the coordinating center and trial steering committee

Vilnius University hospital Santaros Klinikos is the coordinating center of the study, and it will coordinate the trial and trial sites. Bi-monthly meetings led by the principal investigator are held to provide routine organizational support.

A trial steering committee consisting of clinicians (surgeon, medical oncologist), statistician, data manager, and research assistant are established to monitor and supervise the progress of the study. Study monitors will have full access to the data. The monitoring plan includes verification of the informed consent form, checking if patients meet inclusion and exclusion criteria, and monitoring the quality of the data recorded in the case report form. It is planned to review data of the 25% of included patients. Additionally, the steering committee will review relevant information on the topic of the research from other related studies in bi-annual meetings.

#### Composition of the data monitoring committee, its role, and reporting structure

Data monitoring committee (DMC) consisting of clinicians with experience to treat GC patients with PM and to conduct clinical trials will monitor the safety of the trial subjects throughout the study. Safety analyses will be held after each 13 (35%) will complete the assigned treatment. DMC members are independent of the sponsor and will provide a recommendation to stop or continue the study. The advice of the DMC will be shared with the sponsor and principal investigator of the study, who will be responsible to inform the local research ethics committee if necessary.

#### Adverse event reporting and harms

All serious adverse events (SAEs), except those related to the progression of the disease, will be recorded up to 30 days after the last protocol treatment. SAEs will be reported to the principal investigator of the study within 2 working days and to the local research ethics committee that approved the study within 14 days.

### Plans for communicating important protocol amendments to relevant parties

Any changes to the protocol will require formal amendment provided by Vilnius Regional Biomedical Research Ethics Committee.

### Dissemination plans

The trial results will be disseminated to society at national and international conferences and publications in a peer-reviewed journal, irrespective of the study outcomes. Co-authorship will be based on the international ICMJE guidelines.

## Discussion

In this study, we aim to investigate the combination of PIPAC (cisplatin and doxorubicin) and systemic FOLFOX chemotherapy efficacy for the first-line treatment of GC PM.

We designed this study, because of several reasons. First, novel treatment strategies for GC PM are urgently needed as conventional methods (systemic therapy) have only a very limited efficacy with a median OS ranging between 2 and 9 months [2–6]. Innovative drugs, especially immune-checkpoint inhibitors, hold the potential to enhance these outcomes. The phase III CheckMate 649 study demonstrated that the addition of Nivolumab to standard chemotherapy significantly extends the median overall survival from 11.6 (95% CI: 10.9–12.5) to 13.8 (12.6–14.6) months (HR 0.80 (99.3% CI: 0·68–0·94; p = 0·0002) in comparison to standard chemotherapy for treatment-naive patients with gastric, esophagogastric junction, or esophageal cancer [25]. However, it is important to note, that only 23.7% of participants had peritoneal metastases and despite some improvement long-term outcomes remained unsatisfactory.

Second, there is some evidence indicating the potency of PIPAC. A recent systematic review summarized current evidence and suggested that PIPAC can lead to improved long-term outcomes with a median OS of 8-19.1 months [13]. These results are even more encouraging, when the fact that the majority of included patients were already intensively pre-treated with sometimes several different lines of systemic chemotherapy [13], is taken into consideration. Although, current studies have many limitations, including heterogeneity of treatment protocols (PIPAC alone vs. bidirectional treatment) and measured outcomes. Also, the majority of them are retrospective [13]. Thus, there is a need for new prospective phase II studies. Our study experimental protocol consists of PIPAC with cisplatin (10.5 mg/m^2^) and doxorubicin (2.1 mg/m^2^) in combination with FOLFOX as a first-line treatment for GC patients with PM. There is no clear evidence showing the benefits of such bidirectional approach, although, as it is the first-line treatment, systemic control of disease by traditional FOLFOX and additional local (peritoneal) control by PIPAC seems rational and ethically acceptable. Moreover, intraperitoneally applied cytotoxic drugs have only limited penetration to peritoneal lesions of approximately up to 5 mm [26]. Therefore, an intravenously applied cytotoxic drug may have synergistic benefits for peritoneal metastases treatment by affecting tumor nodules from the site of the peritoneal surface [26]. The effectiveness of similar bidirectional approaches has been previously examined, but employing diverse methods for the application of intraperitoneal chemotherapy. In the Japanese phase III PHOENIX-GC trial, the evaluation involved adding intraperitoneal paclitaxel (20 mg/m2) through a peritoneal port or catheter to intravenous paclitaxel and oral S1 for GC patients with PM. The combined intraperitoneal and systemic chemotherapy did not demonstrate a significant improvement in median overall survival (OS) (17.7 months (95% CI: 14.3–21.3 months)) compared to standard systemic chemotherapy (14.8 months (95% CI:12.3–21.8 months)) in the overall study population, as indicated by an HR of 0.72 (95% CI: 0.49–1.04; p = 0.080). However, a post hoc sensitivity analysis, adjusted for baseline ascites, revealed significance (HR: 0.59; (95% CI: 0.39–0.87; p = 0.008)) [27]. Another bidirectional strategy involved laparoscopic hyperthermic intraperitoneal chemopetherapy (HIPEC) after systemic chemotherapy, as reported by Badgwell et al. [28]. In this phase II study, the laparoscopic HIPEC procedure could be repeated up to five times, with 5 out of 19 patients (26.3%) undergoing subsequent radical surgery due to metastasis regression. These patients achieved a median OS of 30.2 months [28]. However, it’s essential to note that in these earlier studies, intraperitoneal chemotherapy was administered without the pressure and aerosolization achieved with the latest technique for intraperitoneal chemotherapy-PIPAC. Compared to conventional methods of intraperitoneal chemotherapy application, PIPAC might offer more uniform drug distribution and improved drug penetration into peritoneal lesions, suggesting potential for enhanced treatment outcomes.

The primary outcome of the present study is objective response rate (ORR) according to RECIST v. 1.1 criteria [20] in a CT scan performed 7 days after the 4th cycle of FOLFOX. RECIST criteria may have limitations when measuring the response to therapy in peritoneal metastases, particularly when the disease burden is minimal. This is because peritoneal metastases can be challenging to determine with standard cross-sectional imaging [29]. However, it’s important to note that our study investigates PIPAC and FOLFOX combination as the first-line treatment for patients with an unresected primary tumor commonly accompanied by lymph node metastases, making the identification of target lesions less problematic. Selecting ORR as the primary outcome is appropriate for a phase II study, aligning with recommendations from the European Society for Medical Oncology, as it allows for the measurement of anti-tumor activity before contemplating a phase III study. Additionally, several secondary endpoints, such as PCI reassessment and histological regression of peritoneal metastases following the 2nd and 3rd PIPAC, are specifically dedicated to evaluating treatment efficacy in peritoneal metastases.

To our best knowledge several other clinical studies investigating bidirectional PIPAC as a first-line treatment of GC PM are currently undergoing (NCT05318794; NCT04913662; NCT05303714). SPECTRA (NCT05318794) single-arm study is investigating the feasibility and safety of 3 cycles of standard systemic chemotherapy interposed with 3 PIPAC (Doxorubicin and Cisplatin) sessions for patients with limited peritoneal disease (PCI ≤ 3) in the United Kingdom. Another phase I study undergoing in South Korea (NCT04913662) investigates dose-limiting toxicity of PIPAC (Paclitaxel) and Systemic FOLFOX combination for GC PM. And finally, there is already a phase III randomized control trial (PIPAC_VEROne; NCT05303714) undergoing in Italy. This study randomizes patients with GC PM to 6 cycles of FOLFOX or 6 cycles of FOLFOX with 3 PIPACs (Doxorubicin and Cisplatin) performed every two cycles of chemotherapy. PIPAC_VEROne study treatment protocol is very similar to the present study. However, different from our study, the Italian trial will include only patients with the limited peritoneal disease (PCI ≤ 6).

Thus, our study will be the first to provide knowledge of PIPAC and FOLFOX efficacy for GC patients with PM, including those with higher PCI scores.

### Trial status

The first patient was included in December 2022. At the time of protocol revision (October 2023) 2 centers in Lithuanian are actively recruiting patients for the study, and 17 patients have already been included.

## Data Availability

The data generated through this study will be available from corresponding author on reasonable request.

## Abbreviations

CR: Complete response
CRFs: Case report forms
CT: Computed tomography
DMC: Data monitoring committee
FOLFOX: Chemotherapy regimen of 5-fluorouracil, oxaliplatin, and leucovorin
GC: Gastric cancer
HR: Hazard ratio
IC: Written informed consent (IC)
ORR: Objective response rate
OS: Overall survival
PCI: Peritoneal carcinomatosis index
PIPAC: Pressurized intraperitoneal aerosol chemotherapy
PM: Peritoneal metastasis
SAEs: Serious adverse events
